# Protoporphyrin IX Beyond Conventional Applications: A Review of Emerging Research Directions

**DOI:** 10.3390/life15101516

**Published:** 2025-09-26

**Authors:** Mustafa Kemal Ruhi

**Affiliations:** Institute of Biomedical Engineering, Boğaziçi University, Istanbul 34342, Turkey; kemal.ruhi@bogazici.edu.tr

**Keywords:** 5-aminolevulinic acid, protoporphyrin IX, photodynamic therapy, photodynamic priming, liquid biopsy, dormant cancer cells, cancer stem cells, neurological and mental health

## Abstract

5-Aminolevulinic acid (5-ALA) is used clinically for photodynamic therapy and fluorescence-guided diagnosis and surgery due to its selective accumulation in malignant cells, where it is converted into photoactive protoporphyrin IX (PpIX) via the heme biosynthesis pathway. The resulting buildup allows for selective visualization or destruction of the tissue under specific light exposure, particularly in pre-malignant and malignant skin lesions, brain tumors, and bladder cancer. More recently, 5-ALA and 5-ALA-induced PpIX have attracted interest for emerging diagnostic and therapeutic approaches. For instance, PpIX is being investigated as a potential marker for liquid biopsy. PpIX-mediated photodynamic therapy also shows promise for targeting specific cancer cell populations, including dormant cancer cells and cancer stem cells. In addition, the benefits of 5-ALA in neurological and mental health are under investigation, as disruptions in heme biosynthesis are increasingly linked to neurodegenerative diseases, chronic fatigue, and mood and sleep disorders. This review highlights these expanding research directions, discusses current challenges, and explores potential opportunities for 5-ALA-based applications.

## 1. Introduction

**Figure 1:**
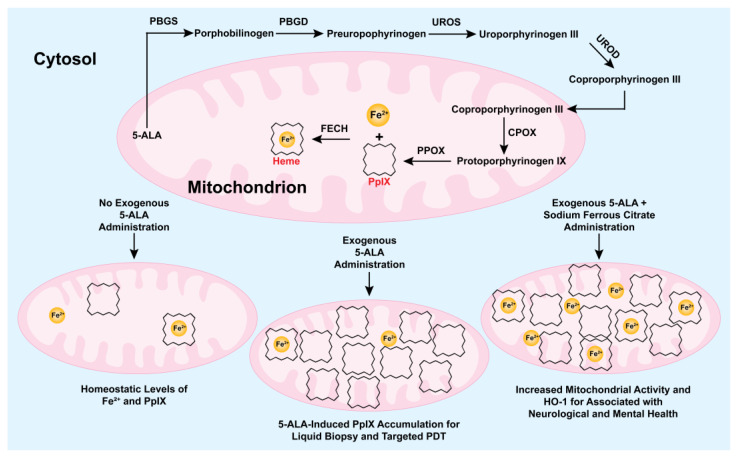
Heme biosynthesis pathway and its role in emerging applications of protoporphyrin IX (PpIX). The top section shows the key steps of the heme biosynthesis pathway, highlighting PpIX (represented as a black serrated ring) and heme (represented as a black serrated ring with Fe^2+^ at its center) production. The branches below illustrate the mechanisms in applications of exogenous aminolevulinic acid (5-ALA), administered with or without ferrous iron, including liquid biopsy; targeted photodynamic therapy of resistant cancer cells; and maintenance of neurological and mental health. HO-1: Heme oxygenase-1, PBGS: Porphobilinogen Synthase, PBGD: Porphobilinogen Deaminase, UROS: Uroporphyrinogen III Synthase, UROD: Uroporphyrinogen Decarboxylase, CPOX: Coproporphyrinogen Oxidase, PPOX: Protoporphyrinogen Oxidase, FECH: Ferrochelatase.

Aminolevulinic acid (5-ALA), a precursor in the heme biosynthesis pathway, has gained significant clinical relevance due to its ability to induce the intracellular accumulation of the photoactive compound protoporphyrin IX (PpIX) [1,2]. Upon administration topically, orally, intravenously, or via newer routes such as instillation or sublingual delivery, 5-ALA bypasses the tightly regulated first enzymatic step of heme synthesis, leading to intracellular accumulation of PpIX (Figure 1) [2,3]. This accumulation is higher in malignant cells due to several tumor-associated factors. Reduced activity of ferrochelatase (FECH) in tumors limits the conversion of PpIX to heme, while increased activity of upstream enzymes such as porphobilinogen deaminase (PBGD) and uroporphyrinogen decarboxylase (UROD) enhances PpIX production [4,5]. Furthermore, limited iron availability, along with changes in 5-ALA import (via peptide transporter 1 (PEPT1)) and PpIX export (via ATP-binding cassette subfamily G member 2 (ABCG2)) that favor PpIX accumulation, contribute to its intracellular retention [5,6,7,8]. Microenvironmental conditions in tumors also play a role in PpIX accumulation. Hypoxia can decrease PpIX accumulation by increasing FECH-mediated conversion of PpIX to heme and upregulating the expression of export transporters for PpIX or its precursor coproporphyrinogen III [9,10]. Conversely, the acidic pH typical of tumors may enhance 5-ALA-induced PpIX formation by increasing PBGD activity [11]. PpIX production is concentration-dependent: increasing 5-ALA availability enhances accumulation, but saturation of enzymatic conversion can limit further PpIX increase [1]. In sum, multiple mechanisms contribute to the preferential accumulation of PpIX in tumors following 5-ALA administration.

5-ALA-induced PpIX production was first identified in the early 20th century through investigations into porphyrin metabolism, and its selective accumulation in diseased tissues after exogenous 5-ALA administration was further characterized in the final decades of the century [12,13,14]. Over the following decades, continued research and optimization of 5-ALA-induced PpIX enabled its clinical translation into two major applications: photodynamic therapy (PDT) and fluorescence-guided diagnosis or surgery [15,16,17,18,19]. 5-ALA-based PDT (5-ALA-PDT) is Food and Drug Administration (FDA)-approved and widely used for the treatment of actinic keratosis, a common pre-malignant skin condition affecting tens of millions worldwide [17,20]. 5-ALA-PDT is also approved in Europe for treating superficial basal cell carcinoma and Bowen’s disease, providing a non-invasive alternative to surgical intervention [21]. In urooncology, hexaminolevulinate (HAL), a hexyl ester derivative of 5-ALA, is approved for fluorescence cystoscopy in the detection of non-muscle-invasive bladder cancer [22,23]. Studies have shown that HAL-guided photodynamic diagnosis increases tumor detection sensitivity compared to standard white-light cystoscopy. For instance, Schubert et al. evaluated two meta-analyses that included 35 studies in total and reported that HAL-guided imaging provides approximately 20% greater sensitivity than white-light cystoscopy, thereby reducing recurrence rates by enabling more complete resections [24]. Another approach for the intraoperative diagnosis of non-muscle-invasive bladder cancer that is approved in Japan is the oral administration of 5-ALA hydrochloride, which similarly enhances tumor visualization and supports more accurate resections [25,26]. Perhaps most prominently, 5-ALA has transformed the field of neurosurgical oncology, where it is used to guide the resection of high-grade gliomas, including glioblastoma multiforme (GBM) [15,16,18]. The administration of 5-ALA, followed by exposure to blue light, enables neurosurgeons to visualize tumor margins in real-time. Clinical trials, such as the phase III study conducted by Stummer et al., demonstrated that 5-ALA fluorescence guidance increased the rate of complete tumor resection from 36% to 65%, significantly improving progression-free survival [19]. As a result, 5-ALA is now approved for fluorescence-guided resection (FGR) of malignant gliomas in multiple regions, including the EU, USA, and Japan, and has become standard practice in many hospitals. To evaluate the accumulated evidence over time, two meta-analyses from the last decade showed that PpIX fluorescence enables intraoperative identification of glioma tissue with reported sensitivities of 82–87% and specificities of ~89% [27,28]. Additionally, a comprehensive systematic review and meta-analysis by Mansouri et al. concluded a diagnostic accuracy of >80% [29]. These performance characteristics underline the clinical value of fluorescence-guided diagnosis and resection.

Building on this clinical success, 5-ALA and 5-ALA-induced PpIX are now being explored for a broader range of applications, both cancer-associated and beyond. One important area of development is the improvement of cancer diagnostic methods, as conventional approaches are often invasive, expensive, and unsuitable for frequent monitoring [30]. In response to this challenge, liquid biopsy has emerged as a minimally invasive technique that detects disease-associated biomarkers in biofluids such as blood or urine, offering a patient-friendly alternative to traditional tissue biopsies. In this context, the use of 5-ALA-induced PpIX levels as a potential liquid biopsy marker has emerged as a rapidly growing research area, offering new possibilities for real-time, non-invasive disease monitoring. Another emerging research field is targeting specific cancer cell populations, such as dormant cancer cells and cancer stem cells, as recent evidence suggests that these populations can contribute to tumor progression and resistance to therapy [31,32]. Accordingly, the targeted photodynamic destruction of these cell populations, or other cancer cells that have developed resistance due to mechanical or chemical stressors, using 5-ALA-induced PpIX is being actively investigated as a standalone or complementary treatment approach. Neurological and mental disorders pose a growing burden on public health, affecting millions worldwide and significantly impairing quality of life [33,34]. As an example, there is currently no clinical cure beyond symptomatic treatment for neurodegenerative diseases (NDs), which are characterized by progressive and irreversible neuronal loss [35]. Studies over the past decade have highlighted the involvement of heme metabolism and mitochondrial function in the progression of these diseases [36,37,38,39,40]. For instance, key enzymes in the heme biosynthesis pathway, 5-aminolevulinate synthase 1 (ALAS1) and PBGD, were found to be reduced in Alzheimer’s disease (AD) patients [41]. Similarly, levels of the heme-containing mitochondrial inner membrane protein complex cytochrome c oxidase (COX) have been shown to decrease in AD [42]. Therefore, restoring dysregulated mitochondrial function and heme metabolism through the administration of the heme biosynthesis precursor 5-ALA is being investigated as a promising therapeutic approach for NDs. Similar mechanisms are being emphasized by researchers exploring the therapeutic potential of 5-ALA for chronic fatigue and sleep disorders, which negatively affect both physical and mental health and are often associated with secondary issues such as depression and hostility [43,44].

**Figure 2:**
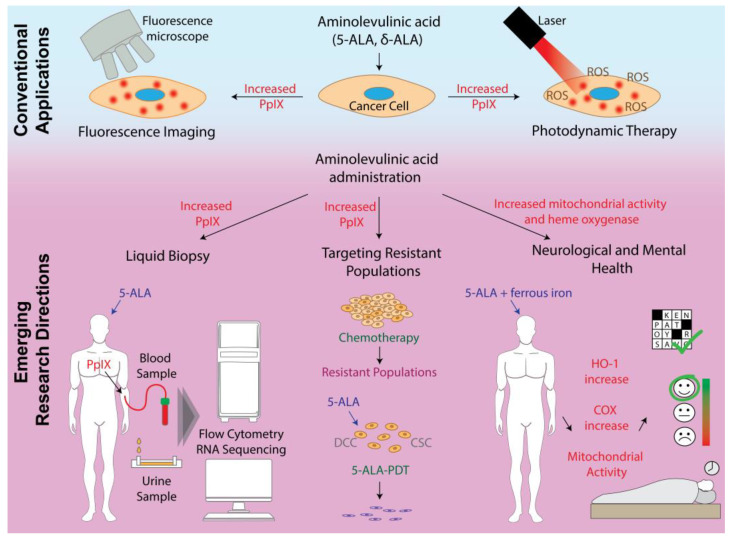
Conventional and emerging applications of 5-aminolevulinic acid (5-ALA) are illustrated on blue and pink backgrounds, respectively. The crossword puzzle, mood chart, and sleeping person on the right-hand side of the figure represent the potential positive effects of the treatment on memory, mood, and sleep. ROS: reactive oxygen species, DCC: dormant cancer cells, CSC: cancer stem cells, HO-1: Heme oxygenase-1, COX: Cytochrome c oxidase.

In summary, 5-ALA is increasingly recognized for its potential in applications beyond its established use in photodiagnosis and PDT (Figure 2). This review explores emerging research directions, outlines key translational challenges, and provides a perspective on possible strategies to address these issues, as well as on directions that may shape future applications of 5-ALA.

## 2. Methodology

This narrative review aimed to summarize emerging applications of PpIX, specifically in liquid biopsy, photodynamic therapy of resistant cancer cell populations, and neurological and mental health. A comprehensive literature search was performed in PubMed, Scopus, and Google Scholar. Publications were retrieved using combinations of the following keywords, without applying any time filters: aminolevulinic acid, ALA, protoporphyrin IX, PpIX, dormant cells, cancer stem cells, resistant, liquid biopsy, mitochondrial activity, neurological health, mental health, Alzheimer’s, and Parkinson’s. Only peer-reviewed articles in English were considered; conference abstracts and non-peer-reviewed journals were excluded. Studies on conventional clinical applications of 5-ALA-PpIX were included only for background information. Titles and abstracts were screened for relevance, and full texts were reviewed to confirm eligibility. References of included studies were also checked to identify additional relevant articles.

## 3. Liquid Biopsy for Glioma

Diagnosis of GBM mostly relies on magnetic resonance imaging (MRI) followed by histopathological evaluation, as no reliable blood-based biomarkers are currently available for this purpose [45]. However, MRI often lacks specificity, particularly in distinguishing actual tumor progression from therapy-related changes, and tissue collection for histopathology may pose clinical risks depending on tumor location [46,47]. Recently, the diagnostic utility of 5-ALA-induced PpIX has expanded beyond fluorescence-guided surgery, with growing interest in its application in liquid biopsy strategies for GBM. For instance, Maas et al. conducted in vitro and ex vivo experiments to isolate and analyze extracellular vesicles (EVs) from U87 malignant glioma cells incubated with 5-ALA, as well as from the blood of glioblastoma patients who received 5-ALA prior to FGR [48]. Using high-resolution flow cytometry, the authors successfully sorted PpIX-containing EVs. Digital droplet PCR further confirmed that these vesicles carried multiple copies of GBM-associated microRNAs, including miR-21 and miR-10b. No consistent correlation was observed between the number of EVs isolated and the amount of detected miRNA, possibly reflecting both technical limitations and the intrinsic heterogeneity of EVs and their cargo. The authors highlighted two major challenges: inter-patient variability in the number of PpIX-positive vesicles and the difficulty of flow cytometry-based detection under low signal-to-noise conditions. Hsia et al. investigated the diagnostic potential of EVs derived from the same cell line used in the previous study (U87) [49]. EVs from U87 cells and primary GBM cell cultures were fluorescently labeled and sorted into five subpopulations (EV_PpIX_, EV_CD9_, EV_CD63_, EV_EGFR_, and EV_CFDA_) using high-resolution flow cytometry and subsequently analyzed for their content of tumor-associated transcripts. Bulk RNA sequencing of these sorted populations revealed that EV_PpIX_ contained the highest proportion of transcripts involved in cancer-associated pathways, including mitogen-activated protein kinase (MAPK) and PI3K-Akt signaling. The authors also analyzed plasma samples collected from GBM patients following 5-ALA administration, which revealed that PpIX-positive EVs were enriched in glioblastoma-associated transcripts, specifically Gremlin 1 (GREM1). Reactome pathway analysis further demonstrated that patient-derived EVs carried cargo significantly enriched in cancer-relevant pathways, including Notch and WNT signaling, oxidative stress-induced senescence, and amino acid metabolism. A clinical study by Walke et al. analyzed systemic accumulation of PpIX in high-grade glioma patients who received 5-ALA before FGR [50]. Blood samples from patients with primary (*n* = 23) and recurrent (*n* = 5) gliomas were collected preoperatively, intraoperatively, and postoperatively, and analyzed using LC–MS. Based on the results, the serum PpIX levels in both primary and recurrent glioma patients were significantly elevated compared to those in healthy controls (*n* = 8), supporting the feasibility of using serum PpIX as a potential diagnostic biomarker for glioma. In another study, Chan et al. developed a microfluidic sensor platform coated with anti-EpCAM (epithelial cell adhesion molecule) antibodies to capture bladder cancer cells from liquid samples, with the ultimate goal of enabling urine-based diagnostics [51,52]. Fibroblasts and bladder cancer cells were cultured and incubated with HAL, and cellular PpIX fluorescence was subsequently imaged under single and double blue light exposures [52]. The method successfully distinguished between cancerous and healthy cells based on fluorescence intensity, with single light exposure recommended to minimize photobleaching.

Together, these studies highlight the emerging potential of 5-ALA-induced PpIX as a functional reporter for minimally invasive cancer diagnosis via liquid biopsy, particularly in GBM and bladder cancer. However, several challenges remain to be addressed before clinical translation can be realized. For instance, the low signal-to-noise ratio in flow cytometry can be overcome by strategies such as careful sample preparation to minimize background noise and enrichment techniques to concentrate vesicles. In addition, using advanced instruments optimized for small particle detection can significantly improve measurement accuracy. Variability in PpIX levels between patients poses another challenge that can be addressed by standardizing 5-ALA dosing and sample collection timing, along with normalization during measurement using complementary techniques such as molecular assays.

## 4. Targeting Specific Cell Types

### 4.1. Dormant Cancer Cells

Growing evidence suggests that dormant cancer cells (DCCs) and cancer stem cells (CSCs), which share overlapping biological pathways and exhibit traits such as quiescence and immune evasion, play a crucial role in tumor initiation, progression, and therapy resistance [31,32,53]. Therefore, developing novel therapeutic strategies that specifically target these resilient populations through mechanisms distinct from standard approaches has become a critical area of cancer research.

5-ALA-PDT works by exciting tumor tissue that preferentially accumulates endogenous PpIX following 5-ALA administration, using ultraviolet or red light to induce the production of cytotoxic reactive species [2]. It has been hypothesized that metabolic alterations in DCCs and cancer stem cells CSCs may lead to a higher selective accumulation of 5-ALA-induced PpIX, potentially enabling targeted elimination of these resistant cell populations through 5-ALA-PDT. Nakayama et al. modeled dormancy by culturing PC-3 prostate cancer cells in 2D and 3D at high cell densities to induce contact inhibition [54]. The authors investigated intracellular PpIX levels, cancer cell sensitivity to 5-ALA-PDT, and the expression of 5-ALA and PpIX transporters following a 24 h incubation with 1 mM 5-ALA. The results confirmed the successful modeling of dormancy in 2D and 3D systems, characterized by reduced expression of the proliferation marker Ki-67 and a decreased number of bromodeoxyuridine (BrdU)-positive cells in a cell density-dependent manner. A decrease in metabolic activity was also evident from reduced intracellular fluorescence of 2-(N-(7-Nitrobenz-2-oxa-1,3-diazol-4-yl)Amino)-2-Deoxyglucose (2-NBDG). Further findings revealed that both the intracellular PpIX and 5-ALA-PDT sensitivity of cells increased in a cell density-dependent manner. This effect was attributed to the intracellular upregulation of PEPT1 and ATP-binding cassette subfamily B member 6 (ABCB6), which facilitate 5-ALA import and intermediate porphyrin transport, respectively, alongside the downregulation of ABCG2, a PpIX exporter, resulting in greater intracellular PpIX retention. Additionally, cells arrested in the G0/G1 phase by agents such as methotrexate and cycloheximide exhibited enhanced 5-ALA-PDT sensitivity, suggesting 5-ALA-PDT’s potential to target DCCs. In a follow-up study, the same group associated elevated PpIX accumulation in DCCs with increased expression of acyl-CoA synthetase (ACS), a key regulator of lipid metabolism [55]. To validate the microarray findings of elevated ACS expression, the authors examined the effects of the ACS inhibitor triacsin C on porphyrin-related transporter expression, observing that triacsin C treatment decreased both intracellular PpIX accumulation and cellular sensitivity to 5-ALA-PDT. A very recent and relevant study by Kasai et al. investigated the relationship between tumor malignancy, cell dormancy, and PpIX accumulation in PC-3 prostate cancer cells [56]. Following a 24 h incubation with 0.3 mM 5-ALA, the authors classified the cells into low and high PpIX-accumulating populations using flow cytometry and analyzed the expression levels of selected proteins. The low-PpIX group exhibited higher expression of receptor activator of nuclear factor kappa-Β ligand (RANKL), a marker of tumor malignancy. In contrast, the high-PpIX group corresponded to dormant cells characterized by reduced proliferation and glucose metabolism, with the former supported by low Ki-67 expression and trypan blue staining results, and the latter evidenced by reduced intracellular 2-NBDG fluorescence. Additional analyses revealed that cells in the low-PpIX group exhibited increased levels of mitochondrial iron, FECH, and heme, suggesting a mechanism in which excess iron is converted into heme rather than accumulating as PpIX. These findings align with the studies discussed above, supporting a link between the metabolic state of cancer cells and intracellular PpIX accumulation.

### 4.2. Cancer Stem Cells

Another cancer cell population often targeted by 5-ALA-PDT is CSCs, which are frequently associated with tumor initiation and treatment resistance [57]. In a recent study by Fujishiro et al., A172 glioma cells were cultured according to a previously established protocol [58] to form glioma stem cell-enriched spheroids, characterized by increased expression of stem cell markers CD133 and SOX2 [59]. Afterwards, the model’s resistance to chemotherapy agents, including cisplatin and paclitaxel, was demonstrated. Subsequently, both the accumulation of PpIX and the 5-ALA-PDT sensitivity of cells were investigated using flow cytometry and a tetrazolium salt assay. Finally, to assess the mechanism of increased PpIX accumulation in glioma stem cells, the expression of enzymes associated with porphyrin transport was evaluated using quantitative real-time PCR. The results showed that the CSC-enriched model, which was resistant to cisplatin and paclitaxel, with a resistance ratio of more than fourfold, exhibited approximately a twofold increase in sensitivity to 5-ALA-PDT performed with a 405 nm laser following a 4 h incubation with 0.3 mM 5-ALA. The authors also reported that each of the assessed enzymes showed increased expression in the glioma stem-like cell-enriched model, specifically PEPT2, ABCB6, 5-aminolevulinate dehydratase (5-ALAD), and protoporphyrinogen oxidase (PPOX), with a fourfold increase. In a subsequent study, the same group evaluated 5-ALA-PDT in five glioma stem cell lines representing mesenchymal (HGG13, HGG30, HGG1123) and proneural (HGG146, HGG157) subtypes [60]. First, the PpIX accumulation and 5-ALA-PDT sensitivity of these cells were assessed. Among the studied cells, those with mesenchymal subtypes exhibited increased PpIX accumulation and 5-ALA-PDT sensitivity, whereas others with a proneural subtype did not. This finding is significant as it underscores the influence of glioma stem cell subtype on 5-ALA-PDT efficacy. The enhanced PpIX accumulation and treatment sensitivity observed in mesenchymal subtypes also suggest that molecular profiling could help identify patients more likely to benefit from this therapy, supporting a more personalized approach to glioblastoma treatment. Further analysis of 5-ALA-PDT-survived HGG13s revealed that these cells exhibited lower stem cell-like properties, as indicated by decreased expression of CD44, Krüppel-like factor 4 (KLF4), and Nestin in PCR, as well as a decreased spheroid-forming ability, characterized by decreased ALDH1A3 expression. Additional studies, not detailed here, have reported similar 5-ALA-PDT efficacy in CSCs from other tumor types [61,62]. However, a recent study by Rice et al., which evaluated CD133 expression as a CSC marker in lung, breast, and colon cancer cell lines, reported findings that contrast with those discussed earlier [63]. Specifically, the study revealed that CD133-positive cell lines were not sensitive to 5-ALA-PDT. Furthermore, DLD-1 colon cancer cells that survived multiple rounds of 5-ALA-PDT exhibited increased expression of the stem cell marker CD133, indicating that 5-ALA-PDT was ineffective in killing these specific cells. However, it should be noted that the high concentration of 5-ALA used in this study (5 mM) might have negatively affected PpIX accumulation and, consequently, 5-ALA-PDT efficacy, as it has been previously reported that intracellular PpIX accumulation does not increase, and may even decrease, at 5-ALA concentrations above 1 mM [1]. Collectively, these findings suggest that, while 5-ALA-PDT holds promise as a therapeutic approach for targeting CSCs, its efficacy may vary depending on the tumor type and the specific PDT protocol used, highlighting the need for further in-depth investigation.

### 4.3. Other Types of Resistance

In addition to the aforementioned populations within tumors, external stimuli, such as exposure to mechanical stress in the tumor microenvironment [64,65,66,67] or chemical agents [68,69], can also induce therapeutic resistance, a phenomenon that has attracted significant research interest. Two recent studies investigated the efficacy of 5-ALA-PDT on ovarian cancer cell lines exposed to fluid shear stress (FSS) and perfluoroalkyl substances (PFAS), respectively [70,71]. In the first study, it was hypothesized that FSS, previously shown to increase resistance to a platinum-based chemotherapy agent, carboplatin [72], could affect 5-ALA-induced intracellular PpIX accumulation and 5-ALA-PDT sensitivity [70]. The two evaluated cell lines, Caov-3 and OVCAR-3, were cultured on regular culture plates (static model) and in ibidi μ-Slide I 0.2 Luer flow chambers under fluid flow of 0.11 mL/min (flow model). The two models were first compared in terms of chemotherapy resistance and 5-PpIX accumulation following 4 h of incubation with 1 mM 5-ALA. Subsequently, the efficacies of 5-ALA-PDT alone and 5-ALA-PDT-mediated priming (5-ALA-PDP) in combination with carboplatin, a platinum-based chemotherapeutic agent, were assessed. The results showed a correlation between the increase in carboplatin resistance and 5-ALA-induced PpIX accumulation in cells, which indicated a potential role of endogenous PpIX as a fluorescent marker for FSS-mediated platinum resistance in ovarian cancer. Both ovarian cancer cell lines cultured under fluid flow conditions exhibited greater resistance to carboplatin and 5-ALA-PDT compared to those cultured under static conditions. Interestingly, under fluid flow, OVCAR-3 cells, which showed greater resistance to carboplatin than Caov-3 cells, accumulated more PpIX and were more sensitive to 5-ALA-PDT. The authors further investigated the effect of priming both static and flow cultures with low-dose 5-ALA-PDT before carboplatin treatment and reported significant decreases in IC_50_ values. Notably, the interaction between the two treatment methods was stronger in the static model, suggesting that fluid shear stress may contribute to cross-resistance between therapies. While these findings reveal the potential of 5-ALA-PDT to overcome FSS-induced carboplatin resistance, they also underscore the need for improved targeting strategies under conditions involving fluid shear stress. In the second study, building on their previous findings that relate PFAS exposure in endometrial cells to platinum resistance [68,73], the authors hypothesized that 5-ALA-PDP (1 mM, 4 h of incubation, 630 nm light irradiation) or benzoporphyrin derivative (BPD)-PDP (0.25 µM, 1.5 h of incubation, 690 nm light irradiation) can overcome carboplatin resistance resulting from short-term exposure to select PFAS [71]. Correspondingly, two HEC-1B and Ishikawa cells grown in culture plates for 24 h were exposed to PFAS or PFAS mixtures for 48 h. The cultures were then treated with either 5-ALA-PDT alone, BPD-PDT alone, carboplatin alone, or primed following the PDT protocols before carboplatin administration. Treatment outcomes were evaluated by assessing mitochondrial membrane potential and the survival fraction of cells. The cells did not show resistance to either PDT protocol. Although BPD-PDP was more effective in inducing mitochondrial photodamage, 5-ALA-mediated PDP more effectively reduced the survival fraction of platinum-resistant cells, suggesting that 5-ALA-PDP may act through additional mechanisms beyond mitochondrial damage.

In summary, all these studies suggest that 5-ALA-PDT or 5-ALA-PDP may offer an effective targeting strategy for various cancer cell populations that could develop resistance to chemotherapy, particularly through the upregulation of enzymes that enhance intracellular PpIX accumulation. However, most of the studies have been limited to in vitro settings, and their efficacy has yet to be demonstrated in vivo. Notably, the hypoxic and heterogeneous nature of tumor tissue poses a risk that these approaches may not be practical in clinical contexts. Therefore, future efforts should focus on optimization of these approaches through in vivo studies or 3D in vitro models that better reflect the tumor microenvironment.

## 5. 5-ALA in Neurological and Mental Health

Heme biosynthesis is a vital cellular process responsible for producing heme, an essential component of hemoproteins involved in oxygen transport, energy metabolism, and cellular respiration [74]. Recently, increasing evidence has linked disruptions in heme synthesis and mitochondrial function to disorders affecting neurological and mental health [40]. To address this link, Omori et al. hypothesized that long-term dietary supplementation with 5-ALA would preserve mitochondrial activity and reduce AD pathology in mice [75]. To test this, triple-transgenic mice were fed 1.8–2.4 mg of 5-ALA daily starting at 3 months of age. After 6 months of treatment, the hippocampus and cerebral cortex were homogenized to evaluate changes in proteins critical to AD progression, including synaptotagmin, COX, mitochondrial membrane potential, and amyloid beta (Aβ), using Western blot analysis. According to the results, COX activity and mitochondrial activity were significantly higher in mice fed 5-ALA. The authors also observed a decrease in Aβ and an increase in synaptotagmin protein levels in the 5-ALA-fed group compared to the controls, indicating a potential reduction in AD-related pathology and improved synaptic function linked to enhanced mitochondrial activity. However, no differences in immunohistochemical analysis using anti-Aβ and anti-pTau antibodies were observed between 5-ALA-fed mice and control animals. A study by Hijioka et al. demonstrated the neuroprotective potential of 5-ALA in rat models of both Parkinson’s disease (PD) and ischemic stroke [76]. In the PD model, where neurodegeneration was induced by injection of 6-hydroxydopamine (6-OHDA) into the substantia nigra, the effect of co-administration of 5-ALA (at doses of 4, 40, or 400 nmol) on rotational behavior was assessed after methamphetamine hydrochloride administration. Subsequently, the rats’ brains were prepared for immunohistochemical analysis to assess the number of tyrosine hydroxylase-positive neurons. The results revealed that 5-ALA administration significantly attenuated motor asymmetry and preserved this neuronal population. In the middle cerebral artery occlusion (MCAO) model of stroke, intrastriatal pre-treatment with 5-ALA reduced infarct volume. Complementary in vitro experiments using human SH-SY5Y neuroblastoma cells further supported the in vivo findings by demonstrating that 5-ALA pre-treatment significantly improved cell viability under conditions of oxidative stress induced by incubation with 50 µM H_2_O_2_. Cells treated with 5-ALA exhibited higher survival rates compared to untreated controls, which was associated with increased heme oxygenase-1 (HO-1) protein expression and reduced reactive oxygen species production, indicating that 5-ALA can mitigate oxidative damage at the cellular level. Another animal study investigating the effect of 5-ALA administration on spatial recognition memory was performed by Komiya et al. [77]. Both intracerebroventricular and oral administration of 5-ALA significantly improved the performance of male ddY mice in the novel object recognition (NOR) test, as revealed through extended exploration periods of new objects compared to familiar ones, and enhanced spontaneous alternation rates in the Y-maze, indicating improved spatial working memory and spatial recognition. According to high-performance liquid chromatography results, treatment with 5-ALA increased the glutamate/Gamma-aminobutyric acid (GABA) ratio, a known indicator of neuronal activity [78], in the dorsal and ventral hippocampus and the entorhinal cortex, regions of the brain involved in recognition memory. At the synaptic level, treatment of hippocampal slices with 5-ALA led to an increase in long-term potentiation (LTP), a crucial process associated with learning and memory. Subsequent experiments revealed that this enhancement was entirely prevented by pre-treatment with 1-naphthyl acetyl spermine, an antagonist that specifically targets calcium-permeable AMPA receptors (CP-AMPARs) lacking the GluR2 subunit, suggesting that the LTP increase following 5-ALA treatment is associated with CP-AMPAR expression.

The potential of 5-ALA was also evaluated in the context of fatigue, mental well-being, and sleep disorders. In these studies, 5-ALA was usually co-administered with sodium ferrous citrate (SFC) to provide ferrous iron, thereby maintaining mitochondrial activity and supporting heme biosynthesis, as illustrated in Figure 1. A randomized, double-blind clinical trial found that 70 adults experiencing chronic fatigue experienced significant reductions in self-reported fatigue and anger–hostility after daily oral administration of 5-ALA phosphate for eight weeks, as measured by the Visual Analog Scale (VAS) and the Profile of Mood States-Second Edition for Adult (POMS2-A) [43]. The 5-ALA group also showed notable improvements in additional mood parameters, including depression and total mood disturbance, compared to the placebo group. The second clinical trial focused on the effects of 5-ALA on sleep and mood in individuals with insomnia-related complaints [44]. A total of 40 participants with sleep disorders between 40 and 70 years old received 50 mg of 5-ALA with ferrous citrate daily for six weeks, followed by a four-week washout period. Results showed that the treatment significantly reduced insomnia severity, as measured by the Pittsburgh Insomnia Rating Scale, within six weeks; however, the benefits tended to diminish after discontinuation. A comparison of the two clinical trials reveals that both highlighted enhanced mitochondrial energy production via increased COX activity as a potential mechanism. The second study also proposed additional pathways, including modulation of neurotransmitters such as serotonin and melatonin, and stabilization of the circadian rhythm. However, none of these mechanisms were directly investigated in either study. A recent case study described a 35-year-old woman with chronic fatigue syndrome who remained symptomatic despite conventional therapies [79]. Treatment with oral 5-ALA/SFC (100–400 mg/day) along with ubiquinone led to marked improvements in daily activity, mobility, and psychosocial function, with no adverse events reported over four years. Genetic analysis revealed a novel heterozygous frameshift mutation in the ADCK1 gene, suggesting a contribution of mitochondrial dysfunction to chronic fatigue syndrome. This case supports the potential of 5-ALA combined with SFC in managing chronic fatigue syndrome.

Together, these studies suggest that 5-ALA supplementation may help prevent neurodegenerative diseases and support mood regulation and sleep quality, potentially through its positive effects on mitochondrial activity, HO-1 expression, and COX expression. However, the findings are constrained by animal studies or clinical trials conducted on small cohorts during short study periods, underscoring the need for further research to validate these effects. Lastly, it is worth noting that, beyond its potential in neurological and mental health, 5-ALA and its co-administration with SFC also show promise in the treatment of metabolic diseases, primarily due to its heme oxygenase-dependent cytoprotective effects and its role in glucose regulation, as supported by a growing body of evidence from both preclinical [80,81,82,83] and clinical studies [43,84]. While the present review does not cover these reports, interested readers are referred to a comprehensive review article that provides an in-depth discussion [85].

## 6. Conclusions and Future Directions

5-ALA is a heme precursor that leads to the selective accumulation of the photoactive compound PpIX in malignant or metabolically active cells. This property has been translated into clinical applications, including PDT and fluorescence-guided surgery or diagnosis, with approval for use in conditions such as actinic keratosis, superficial skin cancers, bladder cancer, and glioblastoma. Beyond these established uses, ongoing research is exploring the broader biomedical potential of 5-ALA, including its applications in liquid biopsies, targeting resistant cancer cell populations, and neurological and mental health.

Liquid biopsy is being investigated as a less invasive and potentially faster alternative to tissue biopsy and certain imaging methods; however, it is not yet advanced enough to replace standard imaging and histopathological evaluations in current clinical practice. The relevant studies discussed here have shown the potential of 5-ALA-induced PpIX as a label for distinguishing cancer cells from healthy cells. Although these studies used advanced techniques such as flow cytometry and liquid chromatography–mass spectrometry, variability in PpIX content and limitations in detection sensitivity remain key obstacles. Future research should focus on optimizing isolation methods, improving detection sensitivity, and validating findings in larger, more diverse patient cohorts to establish robust non-invasive platforms for cancer. The second group of studies discussed in this review converges on the central theme that 5-ALA-induced intracellular PpIX accumulation is sensitive to variations in cellular metabolism and that this metabolic sensitivity could be exploited to selectively target therapy-resistant cancer cell populations with distinct metabolic profiles, such as dormant or stem-like cells. Despite promising outcomes, these strategies remain in the research phase, mainly due to inconsistencies and variability across studies. Indeed, conflicting results, such as the divergent responses of CD133-positive cells to 5-ALA-PDT, underscore the need for further investigations for optimization and mechanistic clarification. The final group of studies covered in this review focused on the effect of 5-ALA on heme biosynthesis and mitochondrial activity, rather than on PpIX accumulation, with the expectation of supporting neurological and mental health in patients. Preclinical studies suggest that 5-ALA may support cognitive function and offer neuroprotective effects, although additional histological data and mechanistic insights would help strengthen its translational relevance. Similarly, early clinical findings point to potential benefits of 5-ALA for mood, fatigue, and sleep quality. Expanding this research through larger and longer-term studies could provide a clearer understanding of 5-ALA’s therapeutic value.

In conclusion, 5-ALA continues to be explored in multiple biomedical applications, from cancer diagnostics and PDT to neuroprotection and metabolic support, offering a promising yet still evolving profile that warrants further investigation across diverse clinical contexts. There is little doubt that the cell-type-specific action of 5-ALA and its effects on metabolism carry substantial potential for other future applications. For example, 5-ALA-PDT may offer a strategy to target another specific type of cancer cell, anoikis-resistant cancer cells, which are known to play a role in metastasis. Also, 5-ALA’s ability to initiate mitochondrial activity can be used to treat other neurological diseases or conditions characterized by altered metabolism, such as hypothyroidism and obesity. Future studies will determine whether the rare and versatile features of 5-ALA can be translated into new diagnostic or therapeutic platforms across a broader range of biomedical fields.

## Data Availability

The data analyzed in the current review are accessible from the corresponding author.

## Abbreviations

The following abbreviations are used in this manuscript:

2-NBDG	2-(N-(7-Nitrobenz-2-oxa-1,3-diazol-4-yl)Amino)-2-Deoxyglucose
5-ALA	5-aminolevulinic acid
5-ALA-PDP	5-aminolevulinic acid-mediated photodynamic priming
5-ALA-PDT	5-aminolevulinic acid-mediated photodynamic therapy
5-ALAD	5-aminolevulinate dehydratase
6-OHDA	6-hydroxydopamine
ABCB6	ATP-binding cassette subfamily B member 6
ABCG2	ATP-binding cassette subfamily G member 2
ACS	Acyl-CoA synthetase
ALAS1	5-aminolevulinate synthase 1
Aβ	Amyloid beta
BPD	Benzoporphyrin derivative
BrdU	Bromodeoxyuridine
COX	Cytochrome c oxidase
CP-AMPAR	Calcium-permeable AMPA receptors
CSC	Cancer stem cell
CPOX	Coproporphyrinogen Oxidase
DCC	Dormant cancer cell
EpCAM	epithelial cell adhesion molecule
EV	Extracellular vesicle
FDA	Food and Drug Administration
FECH	Ferrochelatase
FGR	Fluorescence-guided resection
FSS	Fluid shear stress
GABA	Gamma-aminobutyric acid
GBM	Glioblastoma multiform
GREM1	Gremlin 1
HAL	Hexaminolevulinate
HO-1	Heme oxygenase-1
KLF4	Krüppel-like factor 4
LTP	Long-term potentiation
MAPK	Mitogen-activated protein kinase
MCAO	Middle cerebral artery occlusion
MRI	Magnetic resonance imaging
ND	Neurodegenerative disease
PBGD	Porphobilinogen deaminase
PD	Parkinson’s disease
PDT	Photodynamic therapy
PEPT1	Peptide transporter 1
PFAS	Perfluoroalkyl substances
POMS2-A	Profile of Mood States-Second Edition for Adult
PpIX	Protoporphyrin IX
PPOX	protoporphyrinogen oxidase
RANKL	Receptor activator of nuclear factor kappa-Β ligand
ROS	Reactive oxygen species
SFC	Sodium ferrous citrate
UROS	Uroporphyrinogen III Synthase
UROD	Uroporphyrinogen Decarboxylase
VAS	Visual analog scale
